# Conducive target range of breast cancer: Hypoxic tumor microenvironment

**DOI:** 10.3389/fonc.2022.978276

**Published:** 2022-09-26

**Authors:** Wen Cheng, Xian Xiao, Yang Liao, Qingqing Cao, Chaoran Wang, Xiaojiang Li, Yingjie Jia

**Affiliations:** ^1^ Department of Oncology, First Teaching Hospital of Tianjin University of Traditional Chinese Medicine, Tianjin, China; ^2^ National Clinical Research Center for Chinese Medicine Acupuncture and Moxibustion, Tianjin, China

**Keywords:** hypoxic microenvironment, hypoxia, breast cancer, target, drug resistance

## Abstract

Breast cancer is a kind of malignant tumor disease that poses a serious threat to human health. Its biological characteristics of rapid proliferation and delayed angiogenesis, lead to intratumoral hypoxia as a common finding in breast cancer. HIF as a transcription factor, mediate a series of reactions in the hypoxic microenvironment, including metabolic reprogramming, tumor angiogenesis, tumor cell proliferation and metastasis and other important physiological and pathological processes, as well as gene instability under hypoxia. In addition, in the immune microenvironment of hypoxia, both innate and acquired immunity of tumor cells undergo subtle changes to support tumor and inhibit immune activity. Thus, the elucidation of tumor microenvironment hypoxia provides a promising target for the resistance and limited efficacy of current breast cancer therapies. We also summarize the hypoxic mechanisms of breast cancer treatment related drug resistance, as well as the current status and prospects of latest related drugs targeted HIF inhibitors.

## Introduction

Breast cancer is the cancer type with the highest prevalence, and despite therapeutic advances, still has the second highest cancer-related mortality rate in women (1). One of the main reasons why tumors are difficult to treat is that tumor cells constantly adapt to the adverse environment in which they are exposed. Hypoxia is one of the typical adverse environment, which weakens the function of the tumor. However, malignant tumor cells are often able to compensate for the process of hypoxia and drive the occurrence of later more malignant disease behaviors (2).

Oxygen is essential for energy metabolism, which drives cellular bioenergetics (3). According to Data from a study describing the pretreatment oxygenation status, Oxygen tensions measured in normal breast tissue revealed a mean pO2 of 65 mmHg, whereas in breast cancers of stages T1b-T4, the mean pO2 was 28 mmHg (4). The regions with low oxygen level is generally termed as hypoxic region, which is recognized as a typical microenvironment feature in nearly all solid tumors. Two mainly reasons leading to microenvironment hypoxia can be summarized as follows (1):As most tumor cells are in a state of rapid proliferation and high metabolism, oxygen consumption is far greater than supply, resulting in continuous decline of oxygen content in the microenvironment, and finally formation of hypoxia microenvironment (2, 5).Hypoxia tumor cells secrete vascular endothelial growth factor(VEGF)and other pro-vascular factors to accelerate the regeneration of tumor blood vessels. The density of tumor microvessels was increased, but these vessels were abnormal in structure, which made microvessels unable to regulate blood flow, resulting in hyperperfusion hypoxia (6).

The presence of hypoxic regions is one of the independent prognostic factors for breast and other cancers. Tumor cells, while adapting to hypoxia, lead to more aggressive and therapeutically resistant tumor phenotypes. Hypoxic tumor microenvironment can promote metastasis of tumor cells, inhibits the immune response to tumor cells and changes gene expression, ultimately limiting patient prognosis (7). For example, tumor-associated macrophages (TAMs) have been shown to be associated with poor prognosis of cancer and are predominately localized in the hypoxia regions of tumor. It was found that hypoxia-induced galectin-3 expression and secretion from TAMs promotes tumor growth and metastasis the in orthotopic syngeneic mammary adenocarcinoma model and metastasis model (8).

Considering such changes taken place in hypoxic tumor microenvironment, exploiting for selectively targeting hypoxic areas in breast cancer is an attractive strategy. Some mechanisms of hypoxia leading to drug resistance are being elucidated and drug delivery research has been moving to innovative strategies for breast cancer including engineered nanoparticle based drug/gene delivery systems (9–11). In this review, we briefly discussed microenvironmental changes caused by hypoxia, which are mainly metabolic, genetic and immune levels, and systematically summarized promising advances in targeted hypoxia therapy for breast cancer.

## Hypoxia-inducible factors and breast cancer

The response of cancer cells to hypoxia is principally ascribed to its transcriptional factors HIFs which includes three members, and they are heterodimers composed of an O2 sensitive α subunits (HIF-1α,or HIF-2α,or HIF-3α) and an O2 insensitive HIF-1β subunit (12, 13). HIF-1α is the most well-characterized isoform of the HIFs (14). In normoxiais, it is easily degraded by the ubiquitin-protease hydrolysis complex. Therefore, HIF-1α subunit is virtually undetectable in cells with normal oxygen saturation (15–17). Under hypoxia, degradation of HIF-1α subunit is inhibited and the 1α and 1β subunits form active and stable HIF-1, which is transferred into the nucleus to regulate transcription of multiple genes (18–20). HIF-2α and HIF-3α are two closely related homologues of HIF1α. HIF-1α and HIF-2α share very similar characteristics including their abilities to heterodimerize with HIF-1β, binding to hypoxia-inducible genes and transcriptional activation, but they show different specificity in different tissues and transcriptional targets (21–23). HIF-1α mediated mechanisms favor up-regulation and down-regulation of genes involved in tumor growth and malignant progression as well as epigenetic modification, while HIF-2α stimulates some, but not all, genes activated by HIF-1α. HIF-3α acts as a negative regulator of HIF-1α and HIF-2α mediated gene expression where it can dimerize with HIF-1β and indirectly inhibit HIF-1α and HIF-2α activity (24, 25).

Breast cancer shows extensive clinical and molecular heterogeneity. Prognostic factors are very important for outcome estimation in individual patients. HIF-1 is an important transcription factor in the adaptation of tumor cells to hypoxia, and directly or indirectly regulates cell proliferation and angiogenesis during the progression of tumor hypoxia microenvironment gene expression related to apoptosis and energy metabolism, whose transcriptional activity is a significant positive regulator of tumor progression and metastasis potential (25, 26). Many studies have shown that HIF-1α is overexpressed in breast cancer (27), and HIF-1α has been identified as an independent prognostic factor of breast cancer, and its high expression is significantly associated with poor DFS and OS in breast cancer patients (28–31). A meta-analysis of 5177 patients showed that high HIF-1α expression was associated with high Ki67 expression and strong VEGF expression in advanced breast cancer with lymph node metastasis positive lymph node status negative ER state ductal advanced histological grade (28).In another population-based case-control study evaluating breast cancer recurrence, HIF-1α expression may be associated with early recurrence in patients with ER-breast cancer (32).Additionally, patients with high expression of HIF may be resistant to chemotherapy and endocrine drugs, leading to treatment failure (31, 33).

## The growth and metastasis of breast tumor cells in hypoxic microenvironment

Hypoxia plays an important role in tumor growth and development, related processes include aerobic glycolysis, angiogenesis, immune cells induced to aggregate, and epithelial-mesenchymal transition(EMT).

In the microenvironment of breast cancer, hypoxia activates metabolic changes, from oxidative phosphorylation to a more aerobic glycolytic metabolism (34). Maximum glucose uptake and efficient glucose utilization provide a foundation for glycolysis respiration, thus helping hypoxia cells adapt to the tumor microenvironment, and supporting biological activities such as tumor proliferation, invasion and migration (35, 36). Hypoxia activates transcription factors HIF-1α and FoxO1 and induces epigenetic reprogramming to up-regulate cytoplasmic phosphoenolpyruvate carboxylated kinase (PCK1), a key enzyme that initiates gluconeogenesis, triggering retrograde carbon flow from gluconeogenesis to glycogen decomposition and pentose phosphate pathways. The resulting NADPH promotes the production of reduced glutathione, leading to a moderate increase in reactive oxygen species (37). Tumor stem cells (CSCs) are strongly correlated with tumor progression, metastasis, recurrence and enhanced treatment resistance, and their maintenance of stemness benefits from glycolysis. Peng F et al. found that dehydrogenase kinase 1(PDK1), an important glycolysis enzymes, elevated through the H19/let-7/HIF-1α signal axis, and that downregulation of PDK1 significantly inhibits H19-mediated glycolysis and CSC maintenance. Interestingly, aspirin can significantly attenuate glycolysis and cancer stem-like features by inhibiting H19 and PDK1, providing a potential therapeutic strategy for breast cancer (38). IL-32, known as a pro-inflammatory cytokine, is overexpressed in many types of cancer and enhances tumor cell migration and invasion. Hypoxia-induced reactive oxygen species (ROS) enhances the expression of IL-32β, leading to the activation of IL-32β prolongation of Src, which is involved in the increase of glycolysis and the production of vascular endothelial growth factor (VEGF) under hypoxia (39). Therefore, inhibition of the above targets and pathways may be a therapeutic strategy for inhibiting glycolysis in breast cancer, thereby inhibiting the proliferation and metastasis of tumor cells (40).

Tumor cells grow out of control in tumor tissues, and their internal neovascularization network cannot be established in a timely and effective manner. Therefore, hypoxia controls tumor angiogenesis and malignant progression by regulating the expression of various carcinogenic molecules (41). HIF1α can directly induce the expression of VEGF at the transcriptional level and promote angiogenesis (42). Non-receptor protein tyrosine kinases Syk and Lck play an important role in signal transduction mechanisms of various cellular processes. And their cross-talk regulates hypoxia/reoxygenation (H/R) induces breast cancer progression and further regulates the expression of melanoma cell adhesion molecule (MelCAM) urokinase-type plasminogen activator (uPA) matrix metalloproteinase-9 (MMP-9) and VEGF (43). Immunohistochemistry of 45 patients of breast cancer showed that high levels of HIF1α were positively correlated with increased microvascular density (a measure of angiogenesis) (P=0.023) and with expression of angiogenic growth factors bFGF and PDGF-BB and receptor EGFR (44). Thus, drugs targeting HIF-1 may bind to different pathways that inhibit breast cancer growth, including angiogenesis and growth factors. Tumor-associated immune cells in the hypoxic microenvironment, also play a role in the expression of angiogenesis related signals. HIF-1α/VEGF-A axis is an important pathway for T cells to adapt to the hypoxia microenvironment Analysis of human breast cancer showed that VEGF-A expression was negatively correlated with CD8+ T cell infiltration, and there was a relationship between T cell infiltration and vascular formation (45). TAMs preferentially migrate to the hypoxic region and not only mediate the inhibition of T cells (46), but also directly upregulate angiogenic molecules(VEGF, FGF2, CXCL8, IL-8, type I receptor for VEGF, angiopoietin) or though upregulating of angiogenic modulators (COX2, iNOS, MMP7) to promote angiogenesis (47). In addition, under hypoxia, CAFs can activates VEGF promoters though a transduction pathway formed by HIF1α and its target gene, G-protein estrogen receptor (GPER) (48). Therefore, T cell, TAMs and CAFs play a role in hypoxia-dependent tumor angiogenesis.

Cell culture *in vitro* found that breast cancer cells under the condition of hypoxia training than the cells cultured under the condition of constant oxygen has significant motility (49). EMT is an important biological process for malignant tumor cells to acquire the ability of invasion and metastasis (50). Complete EMT made the epithelial cancer cells transforming into mesenchymal cells and occurring mesenchymal migration or amebic migration. Partial EMT retained the properties of both epithelial cells (cell adhesion) and mesenchymal cells (motility), leading to collective migration of cells, characterized by the presence of leader cells (mixed E/M state) and follower cells (epithelial state) at the front of the invasion, forming the body of the cell population (51). HIF-1α regulated many molecules involved in EMT, for example, HIF-1α regulated TGFβ1/SMAD3 signaling pathway, promoting breast cancer metastasis (52). E-cadherin promoted collective migration of mixed E/M phenotypes by inhibiting TGF-β, while activation of TGF-β leaded to single cell migration (53). Colony stimulating factor 1(CSF-1) played a key role in the control of EMT. Under hypoxia, HIF-1α induced a mixed E/M phenotype through its target gene CSF-1, promoting collective migration (54). Hypoxia leaded to the activation of EMT genes, including TWIST1, SLUG and SNAIL, by degrading PER2, which was considered to be a tumor suppressor, and disrupting the PER2 repression complex (55). X-C Motif chemokine Ligand 1 (XCL1) enhanced expression of HIF-1α and phosphorylation of extracellular signal-regulated kinase (ERK) 1/2, which induces EMT and imposes migration of breast cancer cells (56). Therefore, hypoxia-induced EMT is essential for invasion and metastasis of breast cancer cells. And EMT phenotypes are also associated with stem cell and drug resistance, so further exploration of the molecular details of this process could help develop new therapeutic targets.

## Genomic instability of hypoxia

Intratumoral hypoxia promotes genomic instability, another hallmark of most cancers. It is estimated that up to 1.5% of the human genome is transcriptional responsive to hypoxia (57). In recent years, many genomic changes identified as responsive to hypoxia, may serve as prognostic or predictive markers or even as new therapeutic targets (58). Since increased activity of the HIF-1α pathway is associated with more severe intratumor hypoxia in basal-like breast tumors compared to other subtypes, the gene signature may guide the potential use of future antihypoxia drugs (59–61).

### Hypoxia related DNA

Tumor cells adapt to the hypoxia microenvironment by activating hypoxia-inducible factors to induce the expression of gene products, which are involved in angiogenesis, metabolic reprograming, tumor invasion and metastasis resistance, etc (62). In order to evaluate the changes of hypoxia-induced transcription profile of breast cancer cells, I Chae Ye exposed 31 breast cancer cell lines or normal human breast epithelial cells to either 20% or 1% oxygen. The result showed that in each cell line, more than 1000 genes are induced or inhibited in response to hypoxia, of which 42 genes have conserved responses to hypoxia (63). And all these gene responses under hypoxia were induced by HIF-1α or HIF-2α. Therefore, HIF, as the most important transcription factor in the hypoxic microenvironment, induces a series of changes at the gene level. These hypoxia gene features are meaningful prognostic markers for breast cancer patients and may provide a group of powerful hypoxia treatment targets for the clinic (59, 64, 65).

Studies showed that human breast cancer cells exposed to hypoxia are enough to induce the expression of ADAM12 in a HIF-dependent manner, leading to the shedding of HB-EGF outfield, enhancing EGFR signaling pathway propagation and downstream activation of focal adhesion kinase (FAK) to trigger the breast cancer cells of motility, invasion and metastasis (66). ZMYND8 is acetylated by HIF coactivator P300 in breast cancer cells. And then through the ZMYND8/P300/BRD4/HIF axis, increases angiogenesis, promotes breast tumor progression and metastasis (67). High mobility group box 1 (HMGB1), an important factor in cancer occurrence and development was up-regulated in breast cancer tissues. It regulated hypoxia-inducible factor 1 through the PI3K/AKT signaling pathway, resulting in angiogenesis and tumor migration of breast cancer cells (68). Analysis of previous clinical data shows that basal-like tumors which have the highest rates of metastasis and recurrence are among breast cancer tumors, are associated with higher JFK expression levels and poorer overall survival (69). HIF-1α protein can directly activate JFK transcription, which in turn leads to HIF-1α-induced glycolysis and make hypoxic breast cancer cells insensitive to chemo-radiotherapeutic treatment. In general, the HIF-1α-JFK axis enhances cell tolerance to hypoxia, promotes breast cancer cell survival (70). XBP1 drove TNBC tumorigenicity by regulating the expression of HIF-1α targets through RNA polymerase II recruitment (71). CLDN6 is a tumor suppressor gene for breast cancer. CLDN6, up-regulated by HIF-1α transcription, prevents HIF-1α desulfidation and ultimately leading to HIF-1α degradation through binding the transcription factor β-catenin in the cytoplasm (72).

### Hypoxia related non-coding RNA

MicroRNAs(miRNAs) are endogenous, small non-coding single-stranded RNAs that negatively regulate gene and protein expression primarily by binding to their selective messenger RNAs (mRNAs) (73, 74). Currently, several miRNAs expressed in the hypoxic microenvironment of breast cancer have been identified, which may indicate greater prognostic and therapeutic potential (75). MiR-210 is widely regarded as a powerful HIF target, which is a direct result of decreased oxygen tension in the microenvironment (75, 76). Its expression level in breast cancer samples can be used as an independent prognostic factor (77–79), playing a role in glycolysis, DNA repair, cell survival, immune prediction, chemotherapy resistance, etc. Du Y et al. found that miR-210-3p specifically participated in the Warburg effect (aerobic glycolysis) in TNBC through modulating the downstream glycolytic genes of HIF-1α and p53 (80). In addition, miR-210 inhibits the expression of e-cadherin by targeting the open reading frame region of E-cadherin mRNA and upregulation of e-cadherin transcriptional inhibitor Snail in hypoxic microenvironment, thereby promoting the metastasis, proliferation and self-renewal of breast cancer stem cells (81). Trastuzumab is part of the standard treatment for patients with HER-2 positive breast cancer, but not all patients respond to trastuzumab. An analysis of miRNA expression levels in plasma samples from breast cancer patients showed that circulating miR-210 levels were significantly higher in patients with residual disease than in patients with pathological complete response before neoadjuvant chemotherapy combined with trastuzumab(P =.0359). Therefore, circulating miR-210 level may be associated with trastuzumab sensitivity, tumor presence and lymph node metastasis (82). Chemotherapy resistance is also a serious clinical challenge in breast cancer. MiR-210 regulates JAK-STAT signal transduction pathway by targeting PIAS4, thus affecting the sensitivity of breast cancer to chemotherapy (83).

Most studies on miRNAs in hypoxic microenvironments focus on miR-210, but there are still other miRNAs that respond to hypoxia. Emma Gervin et al. showed that hypoxia can up-regulate miR-655 expression in human breast tumors, which is associated with poor prognosis. In MCF7-miR655 cell lines, the expression of PTEN(negative regulator of HIF-1α) and NFκB1 (positive regulator of COX-2 and EP4) were regulated by down-regulating transcription factors NR2C2, SALL4 and ZNF207, thereby enhancing oxidative stress induced EMT and vascular mimicry (84). In addition, hypoxia and tumor stem cells (CSCs) contribute to paclitaxel (PTX) resistance, the molecular mechanism may be related to miRNA. The experimental data of Liu JH et al. showed that miR-526b-3p attenuates breast cancer stem cell characteristics and chemotherapy resistance by targeting HIF-2α/Notch signaling pathway, which may be used to alleviate chemotherapy resistance in breast cancer (85). MiR-135b may act as a regulatory factor of hormone receptor α(ERα). MiR-135b regulates the protein levels of ERα and HIF1AN by interacting with the 3’UTR region of ERα and HIF1AN (86). Also, miR-153 finely regulated HIF-1α/VEGFA axis by binding to the 3 UTR of HIF1A mRNA, which directly inhibits HIF-1α expression. In this respect, miR-153 can be used for anti-angiogenesis therapy in breast cancer (87).

Long Noncoding RNAs (lncRNAs) are transcripts with more than 200 nucleotides in length but limited protein-coding capacity (88). In the hypoxic microenvironment of breast cancer, some lncRNAs affect the survival and growth of breast cancer cells by regulating HIFs related pathways, providing directions for the possibility of selectively targeted hypoxia therapy (89). TNBC is the most urgent pathological type to be explored, among which three lncrnas are related to hypoxia: IHAT, GHET1 and MIR210HG. LncIHAT promotes the survival of mouse TNBC cells and lung metastasis through the expression of proximal adjacent oncogenes PDK1 and ITGA6 in TNBC cells (90). LncRNA GHET1 leads to over activation of Hippo/YAP signaling pathway, promoting hypoxia-induced glycolysis proliferation and invasion of TNBC (91). MIR210HG directly binds to the 5’-UTR of HIF-1α mRNA, leading to an increase in HIF-1α protein level, thereby upregulating glycolytic enzyme expression (92). In addition to, Zheng F et al. demonstrated that HIF-1α antisense lncRNA HIFAL is essential for maintaining and enhancing HIF-1α mediated retrotranscriptional activation and glycolysis by introducing the PKM2/PHD3 complex into the nucleus. Clinically, targeting lncRNA HIFAL and HIF-1α significantly reduced their impact on tumor growth (93). LncRNA PCAT-1, elevated in breast cancer patients, directly interacts with the activated protein C kinase-1 (RACK1) protein to prevent RACK1 binding to HIF-1α, thereby protecting HIF-1α from RACK1-induced oxygen-dependent degradation of lncRNA (94). Rab11b-as1 enhances the expression of angiogenic factors including VEGFA and ANGPTL4 in hypoxia breast cancer cells by increasing the recruitment of RNA polymerase II, promoting tumor angiogenesis and distant metastasis of breast cancer *in vitro* (95). Hypoxia-induced lncRNA KB-1980E6.3 is abnormally up-regulated in clinical breast cancer tissues. The KB-1980E6.3/IGF2BP1/C-MYC axis maintained the stemness of BCSCs (96). LncRNA NEAT1 is a direct transcription target of HIF-2. It is induced by hypoxia to accelerate the proliferation of breast cancer cells, improve clone survival rate, and reduce apoptosis (97). One of the important mechanisms of lncRNA in hypoxia-related pathways is to antagonize the biological function of miRNA like a sponge (98). LncRNA MALAT1 in hypoxia response can be transcriptionally activated by HIF-1α and HIF-2α, acting as a molecular sponge for miR-3064-5p to promote tumor growth and migration of breast cancer cells (99). LncRNA Vcan-as1 compete with miR-106a-5p, promoting its progression by regulating the miR-106a-5P-mediated STAT3/HIF-1α pathway (100). Phosphoglycerate kinase 1 (PGK1) is an important part of the glycolysis pathway. Zhong Chu et al. found that hypoxia inhibits the expression of LINC00926 which activates the expression of PGK1 mainly through FOXO3A (101). Above, lncRNAs play an important regulatory role in the relevant pathways of breast cancer cells adapting to hypoxia, especially in triple negative breast cancer (102). Therefore, focus on hypoxia related lncRNAs of their potential impact on prognosis and treatment will help predicting new therapeutic agents and exploring mechanisms of drug intervention strategies.

Circular RNAs(CircRNAs) are single-stranded RNA transcripts without 5 caps or 3 polya-tails, but covalently closed ring structures formed by pre-mrna passage and delivery after delivery. CircRNAs mainly target miRNA, act as miRNA sponges, indirectly regulate functional proteins, and participate in cancer progression and hypoxia regulation (103). For example, circDENND4C, which is verified as a sponge for mir-200b and mir-200c, is up-regulated in hypoxia, boosting glycolysis, migration and invasion of breast cancer cells (104). CircRNF20 is highly expressed in BC under hypoxia, through circRNF20/miR-487a/HIF-1α/HK2 axis promoting Warburg effect (105). CircZFR acts as a sponge for miR-578 in BC tissues and cells, promotes the progression of BC malignancy by regulating miR-578/HIF-1α axis (106). Furthermore, Yanxia Zhan et al. screened circRNA differentially expressed between hypoxic and normoxic cancer-associated fibroblasts(CAFs) exosomes by array analysis. The expression of circHIF1A up-regulated in hypoxic CAFs. By which, miR-580-5p has been sponged to modulate dryness of breast cancer cells (107). In addition to competitively antagonizing miRNA, circRNA also has other mechanisms to play a role. CircWSB1 was up-regulated by HIF1α transcription and competitively binds to the deubiquitinase USP10, preventing p53 access to USP10 in BC cells, leading to the degradation of p53 and tumor progression of BC (108). **Table 1**

**Table 1:** Coding and non-coding transcriptome in hypoxic TME.

DNA/RNA	Expression under hypoxia	Signaling pathways	Function	Reference
**DNA**				
ADAM12	Up-regulated	EGFR/FAK signaling pathway	Triggering motility, invasion and metastasis	(66)
ZMYND8	Up-regulated	ZMYND8/P300/BRD4/HIF axis	Angiogenesis	(67)
HMGB1	Up-regulated	PI3K/AKT signaling pathway	Angiogenesis	(68)
JFK	Up-regulated	HIF-1α-JFK axis	Enhancing cell tolerance to hypoxia	(69, 70)
XBP1	Up-regulated	Recruitment RNA polymerase II	Driving TNBC tumorigenicity by regulating HIF-1α targets	(71)
CLDN6	Up-regulated	Binding the transcription factor β-catenin	Leading to HIF-1α degradation	(72)
**miRNAs**				
miR-210	Up-regulated in TNBC	Downstream glycolytic genes of HIF-1α and p53	Activating aerobic glycolysis	(80)
	Up-regulated in BCSC	E-cadherin mRNA	Up-regulating Snail, promoting the self-renewal of BCSC	(81)
	Up-regulated in patients with residual disease	–	Associated with trastuzumab sensitivity	(82)
	Up-regulated	JAK-STAT signaling pathway	Affecting the sensitivity to chemotherapy	(83)
miR-655	Up-regulated	Regulating PTEN and NFκB1 by NR2C2, SALL4 and ZNF207	Enhancing EMT and vascular mimicry	(84)
miR-526b-3p	Up-regulated	HIF-2α/Notch signaling pathway	Alleviate chemotherapy resistance	(85)
miR-135b	Up-regulated	3’UTR region of ERα and HIF1AN	Regulating the protein levels of ERα and HIF1AN	(86)
miR-153	Up-regulated	HIF-1α/VEGFA axis	Angiogenesis	(87)
**lncRNAs**				
IHAT	Up-regulated in TNBC	PDK1 and ITGA6	Promoting the survival of TNBC cells and lung metastasis	(90)
GHET1	Up-regulated in TNBC	Hippo/YAP signaling pathway	Promoting hypoxia-induced glycolysis, proliferation and invasion	(91)
MIR210HG	Up-regulated in TNBC	5’-UTR of HIF-1α mRNA	Upregulating glycolytic enzyme expression	(92)
HIFAL	Up-regulated	antisense RNA of HIF-1α	Enhancing HIF-1α mediated retrotranscriptional activation and glycolysis	(93)
PCAT-1	Up-regulated	RACK1	Protecting HIF-1α from RACK1-induced oxygen-dependent degradation of lncRNA	(94)
Rab11b-as1	Up-regulated	RNA polymerase II	Enhancing the expression of angiogenic factors	(95)
KB-1980E6.3	Up-regulated	KB-1980E6.3/IGF2BP1/C-MYC axis	Maintaining the stemness of BCSCs	(96)
NEAT1	Up-regulated	a direct transcription target of HIF-2	Accelerating proliferation, reducing apoptosis	(97)
MALAT1	Up-regulated	miR-3064-5p	Promoting tumor growth and migration of breast cancer cells	(99)
Vcan-as1	Up-regulated	miR-106a-5P-mediated STAT3/HIF-1α pathway	Activating the STAT3 pathway reversed miR-106a-5p-mediated antitumor effects	(100)
LINC00926	Down-regulated	FOXO3A/PGK1 signaling pathway	Promoting hypoxia-induced glycolysis	(101)
**circRNAs**				
circDENND4C	Up-regulated	mir-200b and mir-200c	Boosting glycolysis, migration and invasion	(104)
circRNF20	Up-regulated	mir-487a/HIF-1α/HK2 axis	Promoting Warburg effect	(105)
circZFR	Up-regulated	mir-578/HIF-1α axis	Boosting malignant progression	(106)
circHIF1A	Up-regulated in CAF	mir-580-5p	Modulating dryness of BC cells	(107)
circWSB1	Up-regulated	deubiquitinase USP10	Leading to the degradation of p53 and tumor progression	(108)

## Hypoxia-mediated immunosuppressive activity

Extreme hypoxia and aberrant HIF-1 activity in the tumor TME are obstacles to effective immunotherapy. In this setting, infiltration and activity of CD8+ T cells are reduced, whereas tumor associated macrophages(TAMs), regulatory T cells(Tregs) and bone marmo-derived suppressor cells (MDSCs) show higher activity. Hypoxic TME also impages cancer-associated fibroblasts (CAFs) and natural killer (NK) cell maturation and activity. Furthermore, hypoxic TME is positively correlated with immune checkpoint expression. These alterations suggest the need for hypoxic regulation as a complementary targeting strategy for immune checkpoint inhibitor (ICI) therapy.

### Innate immunity

Hypoxia can negatively regulate innate antitumor cells in the microenvironment and some key mechanisms. TAMs adopt M1-like proinflammatory phenotypes in the early stages of tumorgenesis and mediate immune responses that inhibit tumor growth. Hypoxia induces the production of a large number of migration stimulators, such as VEGF, EGFR, CCL2, CCL5, CSF-1, oncostatin M, succinate, eotaxin and GM-CSF, produced in the stroma of tumor cells and hypoxic regions (109–112). These stimulators lead to the recruitment of TAM and transformation of M2-like (113), which further promotes its involvement in tumor support processes such as immunosuppressive angiogenesis. Hypoxic TAMs strongly upregulate the expression of REDD1. REDD1-mediated inhibition of mTOR can hinder glycolysis of TAMs and inhibit their excessive angiogenic response, thus forming abnormal blood vessels (114). HIF-1α is a positive regulator of macrophage-derived VEGF. Knockdown the HIF-1α in TAMs attenuates its pro-angiogenic response (115). In addition, it has been recently reported that HIF-1α can up-regulate the expression of PD-L1 in tumor-infiltrating macrophages, thereby promoting the immunosuppressive TME (116, 117).

NK cells are immune cells that kill both virus-infected and tumor cells without antigenic stimulation. The studies of Solocinski and Teng showed that hypoxic stress impaired NK cell cytotoxicity by reducing ERK and STAT3 phosphorylation (118, 119). Ni et al. found that the transcription factor HIF-1α can inhibit NF-KB signaling in tumor-infiltrating NK cells, which is drived by IL-18 to exert antitumor activity (120). However, Seon et al. presented evidence that NK cells stabilized and upregulated their target genes BNIP3, PDK1, VEGF, PKM2 and LDHA by HIF-1α under hypoxia, which activate the ERK/STAT3 pathway to reprogram preactivated NK cells. These reverse the impaired NK effector phenotype and generate necessary number of functional NK cells for adoptive cell therapy (121).

MDSCs have immunosuppressive activity, allowing cancer to escape immune surveillance and not respond to immune checkpoint blockade. HIF-1a enhances the expression of miR-210 in tumor-localized MDSC. MiR-210 regulates Arg1, Cxcl12 and IL16 at both mRNA and protein levels to enhance the immunosuppressant activity of MDSC *in vivo* (122). Deng et al. found that HIF-1α binding to a conserved hypoxic response element in the VISTA promoter, thereby upregulated VISTA in MDSCs. Antibody targeting or gene ablation of VISTA could alleviate MDSC-mediated T-cell inhibition and may mitigate the harmful effects of hypoxia on anti-tumor immunity (123).

**Figure 1:**
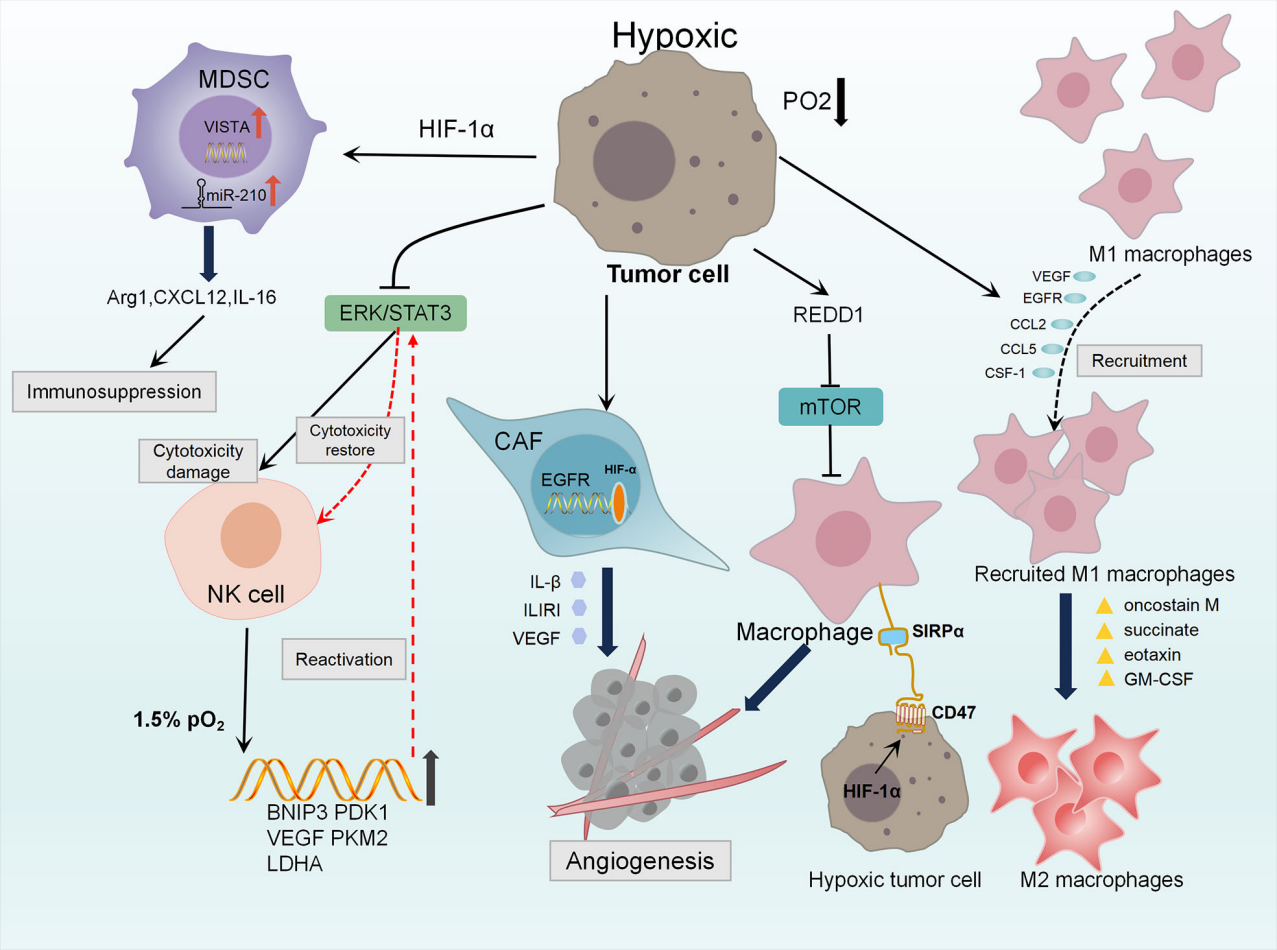
Diagram of the innate immunosuppression in hypoxic TME. Hypoxia induces the production of VEGF, EGFR, CCL2, CCL5, CSF-1 and other stimulators, leading to the recruitment and aggregation of TAMs. Oncostatin M, succinate, eotaxin and GM-CSF polarize M1 macrophages into M2 macrophages which demonstrate tumor-supporting and immunosuppressive functions. Hypoxia strongly up-regulates the expression of REDD1, it could inhibit mTOR to promote abnormal angiogenesis. HIF-1 directly up-regulates CD47, making breast cancer cells escape from macrophage-mediated phagocytosis through CD47-SIRPα axis. Hypoxia up-regulates GPER in CAFs, which is involved in the control of IL1R1, IL-β and VEGF, resulting angiogenesis and invasion of breast cancer cells. Hypoxia damages the cytotoxicity of NK cells by reducing the phosphorylation levels of ERK and STAT3. While Under 1.5% PO2, the ERK/STAT3 pathway reprograms preactivated NK cells through HIF-1α stabilization and higher expression of its target genes BNIP3, PDK1, VEGF, PKM2, LDHA to restore the cytotoxicity of NK cell. HIF-1a increases the expression of miR-210 in MDSC, regulating Arg1 Cxcl12 and IL16 to enhance immunosuppression of MDSC. Also, HIF-1a up-regulates VISTA in MDSCs mediating T cell inhibition.

Stromal fibrosis is a common event in hypoxic TME. CAFs are considered to be the main component of fibrous matrix and can be activated by tumor hypoxia (124). Hypoxia up-regulates the transcription target of HIF-1α, namely G protein estrogen receptor (GPER), that makes CAF-induced IL-1β to express IL1R1 in breast cancer cells (125, 126). HIF-1α/GPER signaling pathway is also involved in the regulation of VEGF expression in breast cancer cells and CAFs exposed to hypoxia (48). Knockdown GPER in CAFs inhibited the invasion of breast cancer cells induced by CAF conditioned medium (125). **Figure 1**

### Acquired immunity

Hypoxic TME inhibits the proliferation and differentiation of CD4+T cells and CD8+T cells mainly by inducing the recruitment and activation of regulatory T cells (T(reg)), initiating autophagy and depletion of T cells, jointly resulted in acquired immune suppression (127, 128). HIF-1 is a key metabolic sensor regulating the balance of T(reg) cells and T(H)17 differentiation. HIF-1 enhances T(H)17 development through tertiary complex formation by recruiting IL-17 promoters with RORγt and P300. At the same time, HIF-1 weakens the development of T(reg) by binding Foxp3 for proteasomal degradation (129). In addition, tumor hypoxia induces the expression of CCL28, CXCL12 and CXCR4, selectively enhanced the recruitment of T(reg) cells, thereby inducing tumor tolerance and new angiogenesis (130–132).

Hypoxia impaired the ability of CD8+T cells in differentiation, proliferation, infiltration and lethality. VEGF-A is the main factor contributor to differential secretion from depleted CD8+T cells under hypoxia. It can promote the differentiation of PD-1^+^TIM-3^+^CXCR5^+^ exhausted-like CD8+T cells and significantly affect the transport and killing ability of CD8+T cells (133). Reports have further shown that anti-VEGF treatment enhances CD8+T cell effector function and provides a mechanistic basis for combining anti-angiogenic and immunotherapeutic drugs in cancer treatment (134). Hypoxia reduces the O2 tension of CD8(+)T cells during activation, upregulates the expression of CD137(4-1BB) and CD25, secrets the immunosuppressive cytokine IL-10. These processes induces the phenotype of CD8+T cells conversing from effector cells to poor proliferation (135).

Hypoxia leads to T cell dysfunction, upon further antigenic stimulation, leads to a state similar to exhaustion. Hypoxia upregulates miR-24 in tumor cells and T cell, both endogenous and exogenous. Mir-24 inhibits the expression of MYC and FGF11 in T cells, thereby disrupting MFN1-mediated mitochondrial fusion. Loss of mitochondrial function generates intolerable levels of ROS, which promotes induction of T-cell exhaustion through phosphatase inhibition (136, 137). Adenosine and adenosine receptors(AR) are important components of hypoxia-related signaling pathways. Hypoxic TME up-regulates the expression of CD39 and CD73. The former is an exonucleoside triphosphate dihydrophosphate hydrolase (ENTPD1) that converts ATP/ADP to AMP. The latter is an exonucleoside 50 enzyme that converts AMP to adenosine (136, 138). Thus, hypoxic adenosine signaling negatively affects T cell activation and effects through adenosine A2A receptor (A2AR), inducing T cell apoptosis (139). At present, preclinical observations have shown that A2AR blockers and immune checkpoint inhibitors cooperate to induce tumor rejection with considerable results (140).

### Role of immune checkpoint blockade

**Figure 2:**
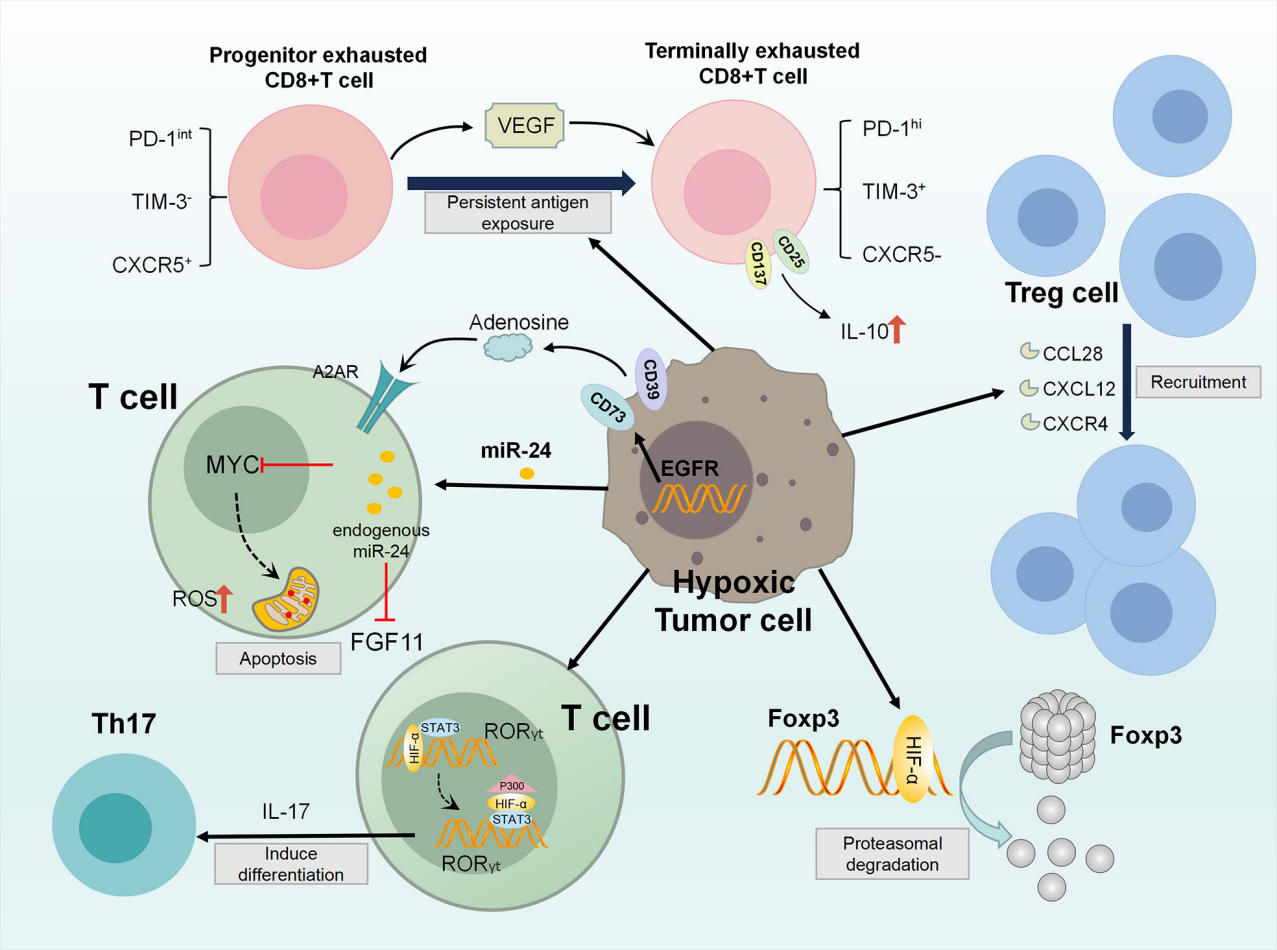
Diagram of the acquired immunosuppression in hypoxic TME. In the hypoxic TME, HIF-1 directly activates RORγt gene transcription in T cells, and then recruits P300 to the RORγt transcription complex to act as the promoter of the TH17 gene (IL-17). These activities promote TH17 differentiation. At the same time, HIF-1 attenuates T(reg) development by binding Foxp3 and targeting T(reg) for proteasomal degradation. Besides, tumor hypoxia induces CCL28, CXCL12, CXCR4 expression, enhancing T(reg) cell recruitment. VEGF-A is a major factor in differential secretion of depleted CD8+T cells under hypoxia, which can promote the differentiation of PD-1^+^TIM-3^+^CXCR5^+^ terminally depleted CD8+T cells. In addition, hypoxia up-regulates the expression of CD137 and CD25, which secretes immunosuppressive cytokine IL-10, eventually inducing adverse T cell phenotype. MiR-24 upregulates in tumor cells and TIL, and inhibits MYC and FGF11 in CD8(+)T cell. Through the destruction of MFN1-mediated mitochondrial fusion, the generation of intolerable ROS levels, causing T cell exhaustion. Further, hypoxic TME up-regulates the expression of CD39 and CD73, which negatively affect T cell activation through adenosine signaling pathway.

Several important immune checkpoints have their own regulatory pathways. In hypoxic TME, almost all of them are directly transcriptional regulated by HIF. In the hypoxic adenosine pathway, CD73 encoded by NT5E gene is a key enzyme for adenosine production and has been considered as a potential immune checkpoint (141). Adenosine receptor has been found in DC, TAM, MDSC and NK cells, implying that adenosine produced by NT5E can inhibit cellular immune responses (142). Thus, NT5E has been identified as a target checkpoint molecule for novel tumor immunotherapy approaches (143). CD47 is an immunoglobulin overexpressed on the surface of cancer cells. CD47 forms a signaling complex with SIRPα expressed on phagocytes and other immune cells, which enables cancer cells to escape macrophage-mediated phagocytosis (144, 145). CD47 is directly regulated by HIF-1 in hypoxic breast cancer cells and plays an immune escape through the CD47-SIRPα axis (146). At present, 23 related drugs targeting CD47 have entered clinical trials and shown good effects (147). MiR-25 and miR-93 are two hypoxic response microRNAs. By targeting NCOA3, they down-regulate the expression of DNA sensor cGAS. This allows hypoxic tumor cells to escape the immune response elicited by the release of mitochondrial DNA, reveals direct link between hypoxic miRNAs and adaptive immune responses to hypoxic tumor microenvironment (148). Programmed death ligand 1(PDL1), which is expressed on the surface of cancer cells, binds to the receptor PD1 on the surface of CD8+T cells, thereby inactivating the antitumor response of CD8+T cells. Hypoxia significantly increases the expression of PD-L1 on MDSCs, TAMs and tumor cells. In addition, the upregulation of PD-L1 under hypoxia depends on the direct binding of HIF-1α to the transcriptional active HRE. Blocking PD-L1 under hypoxia enhances MDSC mediated T cell activation. Therefore, blocking both PD-L1 and HIF-1α may be a promising approach for cancer immunotherapy (116). **Figure 2**

## Hypoxia-induced treatment resistance

Clinical studies have demonstrated that the components in the tumor hypoxic microenvironment are associated with poor prognosis in patients and can promote apoptosis and autophagy or inhibit DNA damage and mitochondrial activity through a number of signaling pathways associated with the failure of immunotherapy, chemotherapy, or radiation therapy (149, 150). This emphasizes that we need to decode the mechanism of hypoxia leading to drug resistance and take measures to promote sensitivity to treatment.

### Hypoxia and radiotherapy

There are good clinical evidences and systematic evaluations that hypoxia is a major negative factor influencing tumor radiation response (150). Preclinical studies in the early 1950s showed that cells can resist radiation damage when oxygen partial pressure is reduced below about 20 mmHg during irradiation (151). Radiation therapy kills cancer by producing ROS, which leads to DNA damage of recipient cells. However, in the case of hypoxia, free radicals produced by DNA under radiotherapy are reduced by molecules containing sulfhydryl group (SH), leading to DNA repair (152, 153). A great deal of efforts have been made to identify ways to overcome radiation resistance caused by hypoxia, including improving the availability of oxygen, increasing the sensitivity of radiotherapy or killing of hypoxia cells to improve the efficacy of radiotherapy.

Hypoxic activated prehaps (HAPs), also known as bioreduction prehaps, are chemically reduced to active compounds at low oxygen levels and target radiation-resistant hypoxic cells. Nevertheless, desirable results have not been achieved in HAPs coupled with radiation therapy (154), possibly due to the failure of the drugs to reach tumor hypoxic areas. Abbasi et al. designed a clinically applicable formulation of mixed manganese dioxide (MnO2) nanoparticles (MDNP) that uses biocompatible materials to react with endogenous H2O2 to regulate TME hypoxia. In a mouse model, approximately 40% of tumor-borne mice were tumor-free after a single treatment of MDNPs plus radiotherapy, 2.5 times lower than the dose required for treatment without MDNPs to achieve the same efficacy (155). A newly prepared single-nanometer oxygen nanobubble water can overcome hypoxia-induced radiation resistance of cancer cells. Under hypoxia, MDA MB231 cells treated with oxygen nanobubble medium significantly inhibited hypoxia-induced HIF-1α and radiation resistance compared with normal medium (156). The upconversion nanoparticle coremesoporous silica shell structure (UCHMs) with the hypoxia activated pro-drug tirapazamine (TPZ) loaded within the cavity between the core and shell could act as excellent delivery vehicles of TPZ to the hypoxic centers of tumors, serve as highly effective radiosensitizer in the meantime, and subsequently kill hypoxic cells during culture. TPZ@UCHMs, this specially designed treatment can also effectively prevent potential hypoxia and reoxygenation, thus effectively inhibiting hypoxia and radiation-induced cell metastasis and tumor regeneration (157). In addition, hyperthermia (heat treatment at 39-45°C) can increase blood flow to improve tissue oxygenation, sensitize radiation through DNA repair inhibition, and can directly or indirectly kill cells by causing vascular damage. This combination therapy has potential clinical applications in the future, but the timing and sequence between radiation and hyperthermia and different action mechanisms caused by heating temperature and heating time need to be further explored (158).

### Hypoxia and chemotherapy

A large number of studies have found that HIF-1 was a necessary condition for chemotherapy resistance of breast cancer stem cells, and HIF-1α expression was correlated with pathological complete response (pCR) in chemotherapy patients (159, 159). Chemotherapy-induced HIF activity accumulated breast cancer stem cell populations through IL-6 and IL-8 signaling pathways and increased expression of multidrug resistance 1 (160). Samanta et al. demonstrated that the combination of HIF inhibitors can overcome breast cancer stem cell resistance to paclitaxel or gemcitabine *in vitro* and *in vivo*, leading to tumor eradication (160). Additionally, hypoxic TME can lead to drug resistance through down-regulation of chemotherapeutic drug targets by HIF-1, reducing the level of topoisomase IIalpha, an enzyme that generates DNA strand breaks when poisoned with etoposide, resulting in chemotherapy resistance of etoposide (161).

Treatment regimen based HIF-1 inhibition has been shown to rescue hypoxia-mediated chemotherapy resistance. Hypoxic-responsive polymeric drug nanoparticles(ICG@CPTNB) release camptothecin CPT by self-combustion in hypoxic regions, significantly improving the tumor growth inhibition efficiency of traditional chemotherapy (162). Based on the high reactivity of manganese dioxide (MnO2) to hydrogen peroxide (H2O2), a bioconjugated manganese dioxide nanoparticles (MAN-HA-MNO2) were targeted to the tumor hypoxia region. It could enhance chemotherapy response by stimulating TAMs to an M1-like phenotype and alleviating tumor hypoxia (163). A hypoxia-activated prodrug can be activated under hypoxia named YC-DOX. It’s self-immolation releases doxoruin (Dox) and YC-1 cysteine, which respectively performs chemotherapy and down-regulates HIF-1α (164).

### Hypoxia and endocrine therapy

About 70% of breast cancer is caused by estrogen through estrogen receptor-α(ERα) (165). Therefore, aromatase inhibitor based endocrine therapy is an important treatment for breast cancer. HIF-1α gene has a typical ER binding element that responds to estrogen signaling, suggesting a direct regulatory link between ERα and HIF-1α pathway in breast cancer (166). Several studies have shown that HIF-1α makes tamoxifen (TAM) resistant to breast cancer cells of ERα+ (167–169). Baicalein helps overcome TAM resistance by promoting the interaction between HIF-1α and PHD2 and pVHL to reduce HIF-1α expression, thereby reducing aerobic glycolysis and reversing mitochondrial dysfunction (168). In addition, hypoxia further down-regulated ERalpha transcription through MAPK signaling and activation of ERK1/2. MEK1/2 inhibitors (U0126 or PD184352) could partially restore ERalpha expression through inhibition ERK1/2. Kronblad et al. demonstrated that U0126 combined with tamoxifen enhanced anti-estrogen effect in hypoxia (169). In a word, the direct and indirect regulatory pathways between ERα and HIF-1α may regulate hormonal responses in endocrine therapy, and it is significant to explore the targets in these pathways for overcoming endocrine resistance and enhancing of efficacy.

### Hypoxia and immunotherapy

Immunotherapy is a promising treatment for triple negative breast cancer (TNBC), but relapse and drug resistance are common (170). Baldominos et al. found that in primary breast cancer, tumor cells resistant to T cell attack are quiescent cancer cells (QCCs). Transcriptomic analysis revealed that QCCs block the function of T cells by regulating the local hypoxic immunosuppressive environment, thus forming a drug library of immunotherapy (171). As described above, adenosine signaling inhibits the activity of T cells and induces apoptosis of T cells through A2AR in hypoxic microenvironment. Inhibition of this pathway plays an important role in improving tumor immunotherapy which mainly through two mechanisms:(a)blocking immunosuppressive adenosine-A2AR mediated intracellular signaling *via* A2AR inhibitors; (b)attenuating HIF-1α mediated extracellular adenosine accumulation by oxygen mixture (142). A2AR blockers, adenosine inhibitors (e.g. CD39 and CD73), as well as hypoxia targeting agents, are currently in clinical phase demonstrated that blocking the hypoxic adenosine-A2AR axis synergistically induces tumor rejection with immune checkpoint inhibitors, providing new hope for the majority of patients who do not respond to immunotherapy (172, 173). Wang Y et al. designed a hemoglobin-poly(Hb-PCL) conjugate self-assembled biomimetic nano red blood cell system(V(Hb)). The V(Hb)@DOX can bind to endogenous plasma haptoglobin (Hp) and specifically target the M2-type TAMs *via* the CD163 surface receptor. The O2 released by the Hb alleviates tumor hypoxia, which further augments the antitumor immune response by recruiting fewer M2-type macrophages (174). In addition, the PFC@lipo modified liposomes can effectively load and release oxygen, helping PD-1 antibody to break through the treatment bottleneck, significantly inhibiting the progression of breast cancer (175). **Table 2**

**Table 2:** Hypoxia-induced resistance related mechanisms and therapies.

Hypoxia-induced resistance	Resistance mechanisms	Therapies	Function	Reference
Radiotherapy	1.Free radicals reduced by molecules containing SH group, leading to DNA repair	HAPs	Chemical reduction to become active compounds that target radiation-resistant hypoxic cells	(154)
		MDNP	Reacting with endogenous H2O2 to regulate TME hypoxia	(155)
		Oxygen nanobubble	Inhibited hypoxia-induced HIF-1α and radiation resistance compared with normal medium	(156)
		TPZ@UCHMs	UCHMs loaded with the hypoxic pre-activation drug TPZ is transported to the tumor hypoxic center, and at the same time serves as a highly effective radiosensitizer	(157)
		Hyperthermia	Increasing blood flow to improve tissue oxygenation, sensitizing radiation through DNA repair inhibition	(158)
Chemotherapy	1. Accumulating breast cancer stem cell populations through IL-6 and IL-8 signaling pathways 2. Increasing expression of multidrug resistance 1 3. Down-regulation of chemotherapeutic drug targets by HIF-1	ICG@CPTNB	Releasing CPT by self-combustion in hypoxic regions	(162)
		MAN-HA-MNO2	Enhancing chemotherapy response by stimulating TAMs to an M1-like phenotype	(163)
		YC-DOX	Releasing doxoruin and cysteine, respectively performing chemotherapy and down-regulating HIF-1α	(164)
Endocrine therapy	1. HIF-1α gene has a typical ER binding element that responds to estrogen 2. Hypoxia down-regulates ERalpha transcription through MAPK signaling and activation of ERK1/2	Baicalein	Overcoming TAM resistance by promoting the interaction between HIF-1α and PHD2 and pVHL to reduce HIF-1α expression	(168)
		MEK1/2 inhibitors (U0126 or PD184352)	Restoring ERalpha expression, enhancing anti-estrogen effect through inhibition ERK1/2	(169)
Immunotherapy	1. Blocking the function of T cells by QCCs 2. Adenosine signaling induces apoptosis of T cells	A2AR blockers(CD39 and CD73)	Blocking adenosine-A2AR mediated intracellular signaling	(172, 173)
		V(Hb)@DOX	Targeting the M2-type TAMs *via* the CD163, releasing O2 and recruiting fewer M2-type macrophages	(174)
		PFC@lipo	Effectively loading and releasing oxygen	(175)

## HIF inhibitors

Targeting the HIF pathway is a direct and effective strategy for alleviating hypoxia in the tumor microenvironment (176). Especially triple negative breast cancer, which has high HIF transcriptional activity but poor response to existing therapies (177). There are two main classes of HIF inhibitors: Direct HIF inhibitors affect the expression or function of the HIF molecule, and indirect HIF inhibitors regulate other molecules in upstream or downstream pathways (such as AMPK, PHD, etc.), ultimately affecting HIF signaling (178). Compared with direct inhibitors, they affect many other pathways, so they are generally less selective for HIF-1α (149). Therefore, direct acting inhibitors of HIF-1α are receiving increasing attention as potential therapeutic agents that specifically target HIF-1 pathways in tumors. Direct HIF inhibitors act through a variety of mechanisms, including inhibiting mRNA expression and inhibiting HIF protein synthesis, affecting heterodimerization of HIF-1α and HIF-1β, inhibiting transcriptional activity of DNA, etc (179, 180). Several promising direct-acting small molecule inhibitors currently under study include: Acriflavone, which can affect HIF-1α dimeration and transcription activation (181), YC-1, Chetomin and Bortezomib, which can inhibit the interaction between HIF-1α and P300/CBP (182–184), and Echinom Ycin and NSC-50352 affect HIF-1α binding to DNA (185). In addition, FIH-1 regulation controls the transcriptional activity of HIF-1α through c-TAD (FIH-1-regulated domain), which is also a potential strategy to target hypoxia-induced malignancy (185). Although many direct inhibitors of HIF-1α have been proposed, none has entered clinical trials. The reasons for their lack of efficacy *in vivo* may be related to the heterogeneity of tumor cells, the complexity of hypoxic microenvironment, and the fact that only HIF-1α targets have been studied while few HIF-2α inhibitors (149). There is still a long way to go before HIF inhibitors can be used in the clinic.

## Conclusion

Hypoxia of the TME in breast cancer and other solid tumors are widespread phenomenon. In response to reduced oxygen tension, HIF1 and HIF-2 stabilize and mediate the hypoxic response, primarily by acting as transcription factors. HIF-1 influences important tumor characteristics, including: cell proliferation, apoptosis, angiogenesis, metabolism, genetic instability and immune response in TME. Therefore, hypoxia mediates resistance to radiotherapy, chemotherapy, endocrine therapy and immunotherapy, and is associated with poor prognosis in cancer patients. The elucidation of this important mechanism of hypoxia also brings new strategies for reversing resistance to current therapies and improving the efficiency of cancer treatment. At present, the main methods for targeting hypoxia are to improve the delivery efficiency by nanocarriers and directly or indirectly inhibit HIF, so as to alleviate tumor hypoxia and prevent HIF from causing tumor support and immunosuppressive effects through a series of signaling pathways. However, these specific targeted hypoxia drugs are still far from clinical practice. In the era of personalized precision medicine, more precise measurements are needed to distinguish between responders and nonresponders to hypoxia-targeted drugs, and more clinical trials are needed to determine whether hypoxia-targeted drugs alone or in combination with existing treatment regimens can increase survival in breast cancer patients.

## Author contributions

WC, XX, and YL designed the manuscript. WC wrote the manuscript. XX and YL drew the figures and tables. QC and CW revised the manuscript. All authors contributed to the article and approved the submitted version.

## Conflict of interest

The authors declare that the research was conducted in the absence of any commercial or financial relationships that could be construed as a potential conflict of interest.

## Publisher’s note

All claims expressed in this article are solely those of the authors and do not necessarily represent those of their affiliated organizations, or those of the publisher, the editors and the reviewers. Any product that may be evaluated in this article, or claim that may be made by its manufacturer, is not guaranteed or endorsed by the publisher.
